# The meaning of actualization of self-care resources among a group of older home-dwelling people—A hermeneutic study

**DOI:** 10.3402/qhw.v8i0.20592

**Published:** 2013-04-19

**Authors:** Ulrika Söderhamn, Bjørg Dale, Olle Söderhamn

**Affiliations:** Centre for Caring Research—Southern Norway, Faculty of Health and Sport Sciences, University of Agder, Grimstad, Norway

**Keywords:** Activity, coping, health, interpretation, qualitative study, social relationship, salutogenesis, self-realization, sense of coherence, well-being

## Abstract

Self-care is an activity of mature persons who have developed their abilities to take care of themselves. Individuals can choose to actualize their self-care abilities into self-care activities to maintain, restore, or improve health and well-being. It is of importance to understand the meaning of the actualization of self-care resources among older people. The aim of this study was to investigate the meaning of the actualization of self-care resources, i.e., actions taken to improve, maintain, or restore health and well-being, among a group of older home-dwelling individuals with a high sense of coherence. The design of this study was to reanalyse narratives revealing self-care activities from 11 (five females and six males) Norwegian older home-dwelling people (65 years or older) identified as having a high sense of coherence. In order to reveal the meaning and get an understanding of why these self-care resources were realized or actualized, a Gadamerian-based research method was chosen. The analysis revealed four themes that showed the meaning of actualization of self-care resources in the study group: “Desire to carry on”, “Be of use to others”, “Self-realization”, and “Confidence to manage in the future”. The findings showed what older people found meaningful to strive for, and this information can be used as a guide for health professionals when supporting older people in their self-care. Older people with self-care resources can also be an important resource for others in need of social contact and practical help. These resources have to be asked for in voluntary work among older people in need of help and, thereby, can be a valuable supplement to the community health care system.

In Scandinavia, it is policy that older people should live at home as long as possible with the goal to live an active and meaningful life in social fellowship with others. Thereby, responsibility lies with the individual person (St. meld. no. 25, 2005–2006). The responsibility is, according to Orem (2001), to take care of oneself in one's own environmental situations. This is self-care, which is an activity of mature persons who have developed their abilities to take care of themselves (Orem, 2001). Self-care can be viewed as a goal-directed activity because the aim is to improve, maintain, or restore health and well-being. To have the capacity to care for oneself is to have self-care ability (Söderhamn, 2000) and, for example, to react on bodily symptoms and be able to change a personal lifestyle or life situation into a healthier one (Söderhamn, 1998a).

Nordenfelt (2009) argues that perceived good health is connected with ability and perceived ill health with disability. Furthermore, Nordenfelt (2009) also states that health, as a bodily and mental state of a person, is often characterized as well-being but also as the person's ability to realize vital goals. Antonovsky (1987) found a person's sense of coherence as an important determinant for maintaining health and for the movement towards a healthy end. Sense of coherence is seen by Antonovsky (1987) as a stress resource oriented concept, which includes comprehensibility, manageability, and meaningfulness that focus on health and problem solving. Dale, Söderhamn, and Söderhamn (2012a) have shown a relationship between self-care ability, perceived health, and sense of coherence in the following way: older persons with higher self-care ability were more likely to perceive good health and to have a stronger sense of coherence than those with lower self-care ability.

Different factors, as described in the literature, influence older home-dwelling people's ability to care for themselves and their health and well-being. According to Kwong and Kwan (2007), perceived self-efficacy has been found to positively influence health-promoting behaviours, such as being physically active and eating a healthy diet. Physical activities have also been highlighted in other studies as an important factor leading to good mental health (Harrison et al., 2010; Windle, Hughes, Linck, Russell, & Woods, 2010), as well as physical health (Harrison et al., 2010). To have positive expectations about health and aging has also been found to be important for older people's perceived health, i.e., when they expect to maintain good health, they are more likely to experience good health (Kim, 2009). Likewise, to have a positive life orientation and close relationships have been found to be important health resources for older people (Fagerström, 2010). To have control over one's own life and participate in social activities and societies also have positive effects on the health status (Chiu & Spencer, 2010). In a study by Söderhamn, Dale, and Söderhamn (2011), older home-dwelling people with a strong sense of coherence experienced self-care as being physically, mentally and socially active. Furthermore, a positive view on life, feeling satisfied with life, being conscious of a healthy diet, and consulting health care professionals when medical problems arise were also considered as issues that influenced self-care positively.

Self-care ability is a necessary condition for self-care activities. It is, however, possible that an individual has this ability but does not make use of it and actualizes it into self-care activity (Söderhamn, 1998b). The meaning of actualizing self-care ability into self-care activities among older people has been interpreted as self-realization and self-transcendence (Söderhamn, 1998a). However, studies among older people that reveal the meaning of the actualization of their self-care ability are very scarce. Little is known about driving forces and motivational issues that lie behind the actualization of self-care abilities into self-care activities. Therefore, new studies have to be implemented in order to understand the meaning of the actualization of self-care resources among older people. This should be valuable knowledge for health professionals in order to support older people in their self-care.

## Aim

The aim of this study was to investigate the meaning of the actualization of self-care resources, i.e., actions taken to improve, maintain, or restore health and well-being, among a group of older home-dwelling individuals with a high sense of coherence.

## Method

The design of this study was to reanalyse narratives revealing issues that influenced self-care activities from 11 older home-dwelling people identified as having a high sense of coherence. The narratives have been analyzed earlier, using a descriptive phenomenological research method (Giorgi, 2009), in order to describe self-care activities among a group of older people (65 years or older) with a high sense of coherence as reported by Söderhamn et al. (2011). In order to reveal the meaning and obtain an understanding of why these self-care activities were realized or actualized, a Gadamerian-based research method (Fleming, Gaidys, & Robb, 2003) was chosen for the reanalysis of the narratives.

According to Gadamer's philosophical hermeneutics (2004), an understanding is only possible through one's pre-understanding. According to Gadamer, everyone has a pre-understanding of the topical question because they are a part of the history (Fleming et al., 2003). The process of understanding described by Gadamer has not been presented as a scientific research method (Debesay, Nåden, & Slettebø, 2008; Fleming et al., 2003). However, based on Gadamer's ideas, Fleming et al. (2003) have outlined the research method used in this study.

### Data collection

The narratives used in this study are derived from informants who participated in a randomized survey study (Dale et al., 2012a) performed in 2010 among older people (65 years or older) living in their own homes in rural areas in southern Norway. In the survey study (Dale et al., 2012a), topics such as health, self-care, sense of coherence, and nutrition were revealed. Fifty-two persons, who were found to have a high sense of coherence, using Antonovsky's (1987) 29-item Sense of Coherence scale, and living in a certain area, were further invited by mail in the autumn/winter of 2010 and the spring/summer of 2011 to participate in the interview study reported by Söderhamn et al. (2011). Of the 52 invited persons, 11 wanted to participate in the interview study and gave their written consent. Of these 11 persons, five were women and six were men and aged between 67 and 89 years (median age 71 years). Background variables of the informants are displayed in Table I. The narratives from these informants were at first analyzed and reported by Söderhamn et al. (2011) and were also the empirical data material in the present study.

**Table I:** Background variables of the informants

Informants	Sex	Age	Civil status
A	Male	74	Widower
B	Female	67	Married
C	Male	67	Married
D	Male	69	Married
E	Female	79	Widow
F	Female	77	Widow
G	Female	71	Married
H	Male	71	Married
I	Male	74	Married
J	Female	71	Married
K	Male	89	Widower

Nine of the informants were interviewed in their homes. One informant wanted to be interviewed in her own office and one in the office of the interviewer. The interviews were opened by the following question: “Please tell me about a situation you have experienced where you could maintain health and well-being.” In order to clarify and elaborate on the informants’ narratives, follow-up questions were used, for example: “Tell me more about that?”, “What did you mean with that?”, and “How do you think about that?”

### The analysis process

The analysis process of the narratives was guided by steps according to Fleming et al. (2003):

1. The analysis starts by reading the whole text in order to obtain an understanding of the content because the meaning of the whole will influence the understanding of the parts.
2. Every sentence or sections were investigated in order to get the text's answer (the horizon of the text) on the meaning of the actualization of self-care resources in order to improve, maintain, or restore health and well-being. Themes were identified that challenged the researchers and the researchers’ pre-understanding of the studied phenomenon (the horizon of the researchers).
3. Every sentence or section was then related to the meaning of the whole text, revealed in the initial phase, and thereby the sense of the text was expanded.
4. Themes that were representative of the shared understanding between the researchers and text, i.e., by fusions of their horizons, were identified.
5. Quotations were used to show the perspectives of the informants in order to establish trustworthiness of the interpretation.

### Ethical approval

The research project about health and self-care among older home-dwelling people in rural areas in southern Norway (Dale et al., 2012a; Söderhamn et al., 2011) have been guided by the Helsinki Declaration (2008) and ethical standard principles (Beauchamp & Childress, 2009), as well as approved by the Regional Committee for Medical Research Ethics in southern Norway (REK Sør-Øst D 2009/1299). Approval was also obtained by the same committee (REK Sør-Øst D 2011/2588) for reanalysing the data collected in the interview study by Söderhamn et al. (2011) using an interpretative method.

## Findings

In the analysis, through the fusions of the horizons by the text and researchers, the following four themes emerged “Desire to carry on”, “Be of use to others”, “Self-realization”, and “Confidence to manage in the future”.

### Desire to carry on

The desire to live was interpreted as a way to cope with challenges. The prioritizing of life gave meaning to actualize self-care resources in order to cope. This self-care was demonstrated in how the informants were conscious of challenges that threatened their health. It could be to understand the relationship between aggravated asthma symptoms and the environment of the work place or to realize that an extensive abuse of alcohol could lead to death. This was a condition for being able to undertake the choice between to live or to die. Life was prioritized ahead of death and, therefore, a strong will to live was guiding goal-directed actions to cope and restore health, as in quitting the job or searching for professional help to become free from alcohol addiction. However, it was crucial to experience support from health professionals and the family to succeed. With this support, it was meaningful to strive to cope with life threatening challenges in order to restore health and well-being and carry on.

I began to get periods of black-outs and I did not know where I was. I then understood that if I did not do something drastic, it was all finished… Yes, but it is not only my merit that I am sitting here today… I have to emphasize that it is with help from the family that I am here today. (Informant A)

The desire to live was also interpreted as having a hope to recover from serious diseases. The hope to get through the disease and recover or maintain a state of well-treated disease could be so strong that another goal was not imaginable. A positive view of life made the hope realistic to reach and thereby it was meaningful to actualize self-care actions. The hope to reach a healthy goal guided the informants in these self-care actions. In order to restore or improve one's own health, such actions were to realize the meaning of adherence to treatment and prescriptions and to be aware of symptoms and know what to do in order to prevent worsening. Furthermore, to continuously be in contact with physicians, to trust fast access if an acute worsening occurred, and to discuss with them about self-care actions provided a feeling of safety and hope of expected effects. To know the physicians well and trust them provided a unique opportunity to freely speak to them about their own efforts. To be accepted for one's own actions was a confirmation of collaboration, but also an affirmation of not being too self-consistent in striving to maintain health and well-being. This provided motivation to continue with self-care actions. This could be to continue to perform job responsibilities, with allowances from the physician, despite a serious disease. This enabled a strong feeling of being better and, thereby, a way to recover. The strong sense of responsibility and that no other person could take over the job, was also a decisive motivator to continue with duties. The choice to continue to work was built on the belief that to do nothing would not lead towards an improvement of the disease.

I think that if you have satisfaction in your job, you will become healthier. I have a chronic disease, and I think it is very important not to sit down… Then I informed the physician that I was working full time and asked if it was a right choice. OK, you are working… I suspected it, he said, and so he took me off the sick list. (Informant B)

When having chronic diseases or disorders, such as diabetes or gluten intolerance that require special diets, it was considered important to follow prescriptions and eat an appropriate diet if one wants to maintain a good quality of life. To have obtained knowledge about the actual disease or disorder and know what to do for managing it provided an understanding of why it was worth an effort. This understanding gave a meaning to realize self-care resources because self-care actions, such as being very careful to eat special diets, were decisive in order to have a more healthy life and also a way to prevent complications or illness and, thereby, to maintain health and well-being.

In order to take care of my health, it is very important for me to be careful with my diet. I am sensing that if I am eating a lot of fruits and vegetables and just a little of other carbohydrates, I can really reduce my diabetes medications. (Informant G)

Moreover, living an active life was interpreted as a life style. To be physically active and to have tasks to do gave life meaning. Likewise, to perform tasks that were considered to challenge the mental capacity, for example crossword puzzles, was seen as an investment in the future. Therefore, both physical and mental activities were considered as necessary self-care actions to maintain health and well-being. Physical outdoor activities, such as taking walks, could be implemented as a way to remove depressive thoughts and prevent stiffness. However, the level of activity was dependent upon the individual's physical condition. Irrespective of the physical condition, an inactive life was not conceivable because it was equivalent to giving up life.

It has been snow and ice the whole winter, but I have been outdoors with my wheeled walker or crutches… I am now outdoors every day and I am walking five kilometers… It is about to be outdoors, and if you are melancholy or you feel unpleasant, then you should take a walk and all such thoughts will disappear. (Informant F)

### Be of use to others

To help other older people was interpreted to be a self-care action to enhance one's health and well-being. Being of use to those who felt lonely, or who needed practical help to manage their daily life, was experienced as a very meaningful activity. To use one's own strengths and resources in favor of others gave so much in return. To be of use to others was engaging and resulted in a feeling of pleasure and happiness. To receive gratefulness for such investments was a source of double joy because it was a reciprocal winning situation. In this sense, to do something for another person was the same as doing something for oneself and, therefore, it was meaningful to use one's own self-care resources to the benefit of others. Being of use to other persons also provides an opportunity for close contacts, which was considered essential to maintain health and well-being.

It is very important to have friends… If you do not have a social network, it is not easy to live… I am visiting a man who is alone. He likes me visiting him, and I like to visit him. (Informant D)

To be useful to others was also experienced as very healthy, as it was a way to remain young and active. It gave an opportunity for one not to be too self-centered, which was considered to be negative for one's own health. To spend time together with the small grandchildren or great grandchildren and doing activities with them was a way to be stimulated and cheered up, but also an opportunity to give service and help to the parents of the small children. Helping others also provided a feeling of being needed and, thereby, still being a resource. This was a strong motivation to actualize self-care resources to use for others and thereby maintaining one's own health and well-being.

Yes, it is something about to be needed. You are not finished. You are not put on the scrap heap. You are still a resource. It means very much. (Informant H)

Having their own experiences of a loss of spouse and children made them especially sensitive to the need for supporting others who had lost their loved ones. To use these experiences and to realize one's own ability to support others was very meaningful because it was possible to use their resources in favor of friends and help them to prevent and alleviate depression and loneliness. Thus, to have friends and to be a friend was a means of being useful for each other that provided strength, health and well-being.

It is very important to be together. It helps very much, indeed, and I will say that we find it very important to get our friends into a normal life after they have lost some of their loved ones. (Informant K)

### Self-realization

Life as a retired person was perceived to be very satisfactory for the informants because it offered them an opportunity for self-realization by doing things they wanted to do or found useful to do. Overall, to do things was considered important and an investment in order to maintain one's own health and well-being. The freedom to choose what to do was a unique possibility for self-realization. This freedom could also be to continue with their work due to the fact that their competence was sought after. To be needed in working life or to be acknowledged for efforts were motivating for continuing the job. Another possibility for self-realization was to look forward and meet new challenges, such as to take courses and learn new skills. This self-development was meaningful in order to maintain health and well-being. To look back and write memoirs in order to share experiences from the old days with grandchildren and great grandchildren was a way to give the family a place in history that give a feeling of being of use to the new generations.

For me it is very important to have something to deal with, something I am engaged in. I have always wanted to do new things. And I have not given it up still because I am looking for some courses to do. (Informant H)

It was also highly appreciated and motivating for the informants to search for such types of relationships where they could be accepted and could be the persons they actually were. This was a self-realizing action that represented health and well-being. A flexible holiday together with some friends or relatives, where they had the possibility to choose between being together and being on one's own, was very positive for a person who valued and longed to have some time by himself or herself. On the other hand, to travel together with other people who had the same interests could be very fulfilling.

We were 16 persons that walked the pilgrim tour. They were very nice people, and we had a pleasant time…and when you know them, you can totally relax. (Informant H)

To enjoy life and be grateful and satisfied for being able to manage daily life and for having an active social life were acknowledged as influencing good health and well-being. They experienced having a good life when starting a new day and having the opportunity to fill it up with activities and being together with other people, or just taking the days as they came. To find a balance between performing activities and taking a break was important because a stressful life was devalued. To strive for such a life was unfolding and, therefore, there was a motivation to continue with activities and actions that promoted this good life and thereby a way to maintain health and well-being.

Imagine thinking every morning: What will I do today? It is excellent! (Informant J)

### Confidence to manage in the future

Having experienced tough periods in life and managing to live through them gave the informants a strength and belief to manage future challenges as well. Being confident that they can cope with challenges in the future gave them a feeling of safety on which to dwell. To achieve this insight was a process to develop mental maturity that can be interpreted as self-care directed to the future. In this process, it was also necessary to realize that life has an end and accept the life situation in order to go further.

When I got cancer I thought it through some days. So I was finished with it. I saw on it with a good mood. The more you have gone through, the more it will help for the future. (Informant D)

Having a strong desire to live in their own dwelling as long as possible was motivating for investing in health and well-being for the future. Moving to an adjusted dwelling in order to be able to live in one's own home, despite difficulties of moving, was an example of such a self-care action. Being satisfied with the dwelling and a feeling of being attached and connected to their residence gave a feeling of safety for the future as well. However, despite a positive view of the future, some of the informants worried about growing older and not being able to manage daily life because of cognitive decline. If this should be a reality, living in a nursing home was seen as a possible solution. To realize that such an event could be possible and having a plan for the future was to perceive health and well-being because they had faith in being cared for in the future. For those who were Christians, the faith in God resulted in gratefulness for life and a feeling of peace because whatever happens, life is in the hands of God. The belief in God was thereby a way for maintaining health and well-being in the future.

I mean that we, who are Christians, are very lucky…we do not need to think negative thoughts because we can slough them and get a new start and look forward… Yes, you have to put the future in the hands of God. (Informant G)

## Discussion

The aim of this study was to investigate the meaning of the actualization of self-care resources, i.e., actions taken to improve, maintain, or restore health and well-being, among a group of older home-dwelling individuals with a high sense of coherence. The findings revealed that the informants found it meaningful to actualize self-care resources because they had a desire to live and to carry on in life and to realize themselves. It was also meaningful for them to use their self-care resources for being of use to others. Moreover, they had the confidence to manage future challenges in life. From these findings, three types of self-care can be outlined, i.e., care for self, care for others, and care by others. These three types of self-care have also been presented by Godfrey et al. (2011). This can be applied to the findings in the present study as follows: To care for self was to take responsibility for one's own health and actualize resources to improve, maintain, or restore health by, for example, striving for coping with a disease or choosing to practice an active lifestyle. To care for others was to be aware of other peoples’ loneliness or needs of practical help and to support and help them. Care by others can be applied when medical help was searched for and carried out partly by others, often in collaboration with the older person. However, this type of self-care, i.e., care by health professionals, has been described by Orem (2001) as compensatory care due to declined ability for self-care.

The findings support Antonovsky's (1987) theory of sense of coherence, with the three components of comprehensibility, manageability, and meaningfulness, that imply that one person with a high sense of coherence will find things understandable and have resources to manage challenges and find it meaningful to use the resources in order to cope and move to a healthy end. A good illustration of this theory was the informant who desired to prioritize life and realized that his alcohol abuse was dangerous and, therefore, found it meaningful to collaborate with others in order to cope with the problem and restore health and well-being.

The desire to live and carry on was shown, for example, by realizing a threatening challenge and to find it worthy to use self-care resources in order to cope and, thereby, restore health and well-being. This shows that self-care is goal-directed (Orem, 2001; Söderhamn, 2000) and that the informants found it meaningful to actualize their self-care ability in order to restore health.

To have a hope to recover from a disease also gave meaning to actualize self-care resources by, for example, following prescriptions and being aware of symptoms in order to not get worse. Arvidsson, Bergman, Arvidsson, Fridlund, and Tops (2011) reported similar findings in a study among a group of both younger and older people with rheumatic diseases. This group experienced that their self-care was guided by a hope of improvement. The present findings also showed that a positive view of life made the hope realistic to reach. This emphasizes a positive life orientation as an important health resource (Fagerström, 2010).

To live an active life was motivating for the informants for maintaining both physical and mental health. To be physically active and have tasks to do gave life meaning. Supporting results have also been found in some quantitative studies. For example, Harrison et al. (2010) could show an association between better-perceived health and increased physical activity in older people, and Windle et al. (2010) found that exercise had a positive influence on mental well-being in older age. In a qualitative study (Sundsli, Espnes, & Söderhamn, 2013), physically active older persons expressed that being physically active as long as possible not only improved and maintained health but could also positively influence longevity. The present findings also showed that it was meaningful to remain active by performing physical activities outdoors because this could lead to reduced stiffness. It is interesting that Arvidsson et al. (2011) reported corresponding findings in their study. Their informants experienced satisfying outcomes of a self-care activity in order to get a lower degree of stiffness, which provided confidence regarding their ability to perform such health-promoting activities.

Being of use to others was a meaningful activity in order to maintain health and well-being because it was beneficial for others. According to Sundsli et al. (2013), to make one useful for others is beneficial self-care. Moreover, being of use to others also represented opportunities for social interactions with others in the present study. This is supported by Dale, Söderhamn, and Söderhamn (2012b), who found in a study among a group of single living older people, that social relationships were of importance for quality of life because it included feelings of being of use to others, in addition to being valued by others. It was also found in the present study that being together with grandchildren and great grandchildren was a way to be stimulated and cheered up. According to Arai et al. (2007), social interactions within the society, neighbors, family, and friends are of importance for preventing depressive moods. Furthermore, Chiu and Spencer (2010) found that living an active social life have positive impacts on older people's health status. In the present study, the feeling of being needed was also found to be a motivator to actualize resources to help others. Close relationships and feelings of being needed in old age are important and should, therefore, according to Fagerström (2010), be a focus in health promotion of older people. This indicates that older people can be a valuable resource in voluntary work for other persons who are in need of practical help and social contact.

To be a retired person provided opportunities for self-realization because the freedom to choose to do things one enjoyed or found useful to do was a way to obtain health and well-being. This was also found to be highly appreciated in another group of people who looked forward to a coming life as pensioners because retirement was related to freedom and choices about what to do (Söderhamn, Skisland, & Herrman, 2011). To have this type of freedom could also result in the choice to continue work instead of being retired, according to the present study.

To search social activities that gave the informants the possibility to be the persons they were was highly appreciated. It could be the feeling to relax in the company of others when performing activities together, and it could also be the possibility to choose when social interaction was desired or not. Similar findings were reported by Dale et al. (2012b), i.e., despite it being very important to have close relationships, solitude was also highly valued. In the study by Sundsli et al. (2013), an inclusive and supportive fellowship was emphasized by the older informants. Such fellowship promoted self-care and health.

The informants had confidence in their own future, despite realizing the possibility for being dependent upon other people's care. To have managed tough experiences earlier in life made it meaningful to have confidence to manage future challenges. To have such a positive view of life will represent an inner health resource, according to Fagerström (2010). The informants had a high sense of coherence, which also will provide them inner strengths to cope with challenges and confidence to manage in the future. Bowling and Iliffe (2011) found, in a quantitative study, psychological resources in older people to be crucial for healthy ageing. To have self-efficacy, resilience, optimism, confidence, and experiences of coping with difficulties were examples of psychological resources that can determine a successful aging. The findings in our study can be seen to illustrate the results in the study by Bowling and Iliffe (2011). To summarize, our informants’ inner strengths, optimism, and experiences of coping with difficulties gave them the confidence to actualize self-care resources to manage challenges in the future. To have faith in being able to take care of oneself or to be cared for by others in the future can be examples of achieved resilience.

### Methodological considerations

There are some weaknesses and limitations in this study. It would have been preferable to have more informants with an advanced age. The informants had a rather low median age and, therefore, the findings to a higher degree revealed actualized self-care resources of younger older people. However, the findings represent all of the informants’ experiences and the oldest of them was 89 years old.

The findings are the result of a reanalysis of narratives, which may be seen as a possible weakness. According to the Gadamer-based research method (Fleming et al., 2003) used in this study, it is fundamental with a dialogue to obtain an understanding of the phenomenon studied. This dialogue can begin during the interviews. However, a reanalysis of narratives provides no additional access to the informants. The first author performed the interviews and, thereby, has first-hand knowledge from the informants that can be an advantage in the interpretation process. On the other side, Austgard (2012) argues that when using a Gadamer-based hermeneutic research method it is most important to understand the text. The text was the starting point for the interpretation in this study.

To assure the trustworthiness of the interpreted findings is a challenge when using a hermeneutic research method. To use quotations gives the reader an opportunity to judge the interpretations (Austgard, 2012; Fleming et al., 2003). Moreover, the interpretations are also a result of the interpreters’ pre-understanding, and it is possible to gain more than one interpretation. Therefore, it is important that the authors’ pre-understanding is disclosed. The researchers’ pre-understandings are that they are nurses and researchers with experiences of nursing older people and carrying out research studies among older home-dwelling people with a focus on self-care and health. However, in the interpretation process, the interpreters have to be especially aware of the text and how they use their pre-understanding, as the interpreters’ pre-understanding can also bias the text (Austgard, 2012). This has been given attention in the present study.

By reflecting about our pre-understanding and challenging our knowledge of the field, we have tried to go beyond the actual interview text. We mean that a consensus between the parts and the whole of the text has been achieved and that the fusion of horizons has been established in the four themes that may be considered as motivational factors for actualizing self-care ability into self-care activity in this group of older people with a high sense of coherence. In particular, the social dimension of self-care challenged our pre-understanding and is in a way a result that should be emphasized on both an individual and societal level when considering care of older people.

The presented interpretation is one of many, but the rigor is partly confirmed through the agreement with a number of other studies, both qualitative and quantitative, among older people. Therefore, it also seems reasonable that the findings have relevance for other older home-dwelling people with self-care resources and could be transferred to such groups in similar contexts.

## Conclusions

The findings showed that the study group found it meaningful to use their self-care resources. Motivating factors for actualization of these self-care resources, in order to improve, maintain, or restore health and well-being, were the desire to carry on and having to cope with challenges. Moreover, the possibility for self-realization was the freedom to choose meaningful things to do. Being of use to others was especially meaningful as it gave the feeling of being needed and to be a resource for others, and provided rich opportunities for close relationships. Another factor that was decisive for the actualization of self-care resources in the future was the confidence to manage future challenges.

The findings showed what older people found meaningful to strive for in order to improve, maintain, or restore health and well-being and this information can be used as a guide for health professionals when supporting older people in their self-care. Health care professionals should be aware of the resources among older people and support them to value these assets and promote their beliefs in themselves and the future and also in the great importance of social relationships. Older people with self-care resources can also be valuable for others in need of social contact and practical assistance. For example, these resources have to be asked for in voluntary work among older people in need of help and, thereby, become a valuable supplement to the community health care system.
